# Author Correction: Cell volume controlled by LRRC8A-formed volume-regulated anion channels fine-tunes T cell activation and function

**DOI:** 10.1038/s41467-023-44400-x

**Published:** 2024-01-05

**Authors:** Yuman Wang, Zaiqiao Sun, Jieming Ping, Jianlong Tang, Boxiao He, Teding Chang, Qian Zhou, Shijie Yuan, Zhaohui Tang, Xin Li, Yan Lu, Ran He, Ximiao He, Zheng Liu, Lei Yin, Ning Wu

**Affiliations:** 1 Department of Immunology, School of Basic Medicine, Tongji Medical College, Huazhong University of Science and Technology, Wuhan, China; 2 State Key Laboratory of Virology, Hubei Key Laboratory of Cell Homeostasis, College of Life Sciences, Renmin Hospital of Wuhan University, Wuhan University, Wuhan, China; 3 Department of Traumatic Surgery, Tongji Trauma Center, Tongji Hospital, Tongji Medical College, Huazhong University of Science and Technology, Wuhan, China; 4 Department of Physiology, School of Basic Medicine, Tongji Medical College, Huazhong University of Science and Technology, Wuhan, China; 5 Medical Research Center, Guangdong Provincial People’s Hospital, Guangdong Academy of Medical Sciences, Guangzhou, China; 6 Department of Clinical Immunology, The Third Affiliated Hospital of Sun Yat-sen University, Guangzhou, China; 7 Department of Otolaryngology-Head and Neck Surgery, Tongji Hospital, Tongji Medical College, Huazhong University of Science and Technology, Wuhan, China; 8 Cell Architecture Research Center, Tongji Medical College, Huazhong University of Science and Technology, Wuhan, China; 9 The First Affiliated Hospital of Anhui Medical University, Institute of Clinical Immunology, Anhui Medical University, Hefei, China

**Keywords:** Cell signalling, CD8-positive T cells, CD4-positive T cells, T-cell receptor

Correction to: *Nature Communications* 10.1038/s41467-023-42817-y, published online 04 November 2023

The affiliation of Lei Yin was incorrectly stated on the published manuscript as “Department of Physiology, School of Basic Medicine, Tongji Medical College, Huazhong University of Science and Technology, Wuhan, China”. The correct affiliation is as follows “State Key Laboratory of Virology, Hubei Key Laboratory of Cell Homeostasis, College of Life Sciences, Renmin Hospital of Wuhan University, Wuhan University, Wuhan, China”.

This has been corrected in both the PDF and HTML versions of the Article.

