# Supplementary material for: Cell volume controlled by LRRC8A-formed volume-regulated anion channels fine-tunes T cell activation and function
Source: Nat Commun. 2023 Nov 4;14:7075. doi: 10.1038/s41467-023-42817-y (PMC10625614; doi:10.1038/s41467-023-42817-y)
Supplement: Supplementary file 1 — Supplementary Information [file 41467_2023_42817_MOESM1_ESM.pdf]

SUPPLEMENTARY INFORMATION

Cell volume controlled by LRRC8A-formed volume-regulated anion channels controls T cell activation and function

Yuman Wang[1], Zaiqiao Sun[2], Jieming Ping[1], Jianlong Tang[1], Boxiao He[2], Teding Chang[3], Qian Zhou[4], Shijie Yuan[1], Zhaohui Tang[3], Xin Li[5], Yan Lu[6], Ran He[1], Ximiao He[4], Zheng Liu[7,*], Lei Yin[4,*], and Ning Wu[1,7,8,9,*]

[1]Department of Immunology, School of Basic Medicine, Tongji Medical College, Huazhong University of Science and Technology, Wuhan, China

[2]State Key Laboratory of Virology, Hubei Key Laboratory of Cell Homeostasis, College of Life Sciences, Renmin Hospital of Wuhan University, Wuhan University, Wuhan, China

[3]Department of Traumatic Surgery, Tongji Trauma Center, Tongji Hospital, Tongji Medical College, Huazhong University of Science and Technology, Wuhan, China

[4]Department of Physiology, School of Basic Medicine, Tongji Medical College, Huazhong University of Science and Technology, Wuhan, China

[5]Medical Research Center, Guangdong Provincial People's Hospital, Guangdong Academy of Medical Sciences, Guangzhou, China

[6]Department of Clinical Immunology, The Third Affiliated Hospital of Sun Yat-sen University, Guangzhou, China

[7]Department of Otolaryngology-Head and Neck Surgery, Tongji Hospital, Tongji Medical College, Huazhong University of Science and Technology, Wuhan, China

[8]Cell Architecture Research Center, Tongji Medical College, Huazhong University of Science and Technology, Wuhan, China

[9]The First Affiliated Hospital of Anhui Medical University, Institute of Clinical Immunology, Anhui Medical University, Hefei, China

*Correspondence: wuning118@gmail.com (N. W.), yinlei@whu.edu.cn (L. Y.), or zhengliuent@hotmail.com (Z. L.)

Supplementary Figures

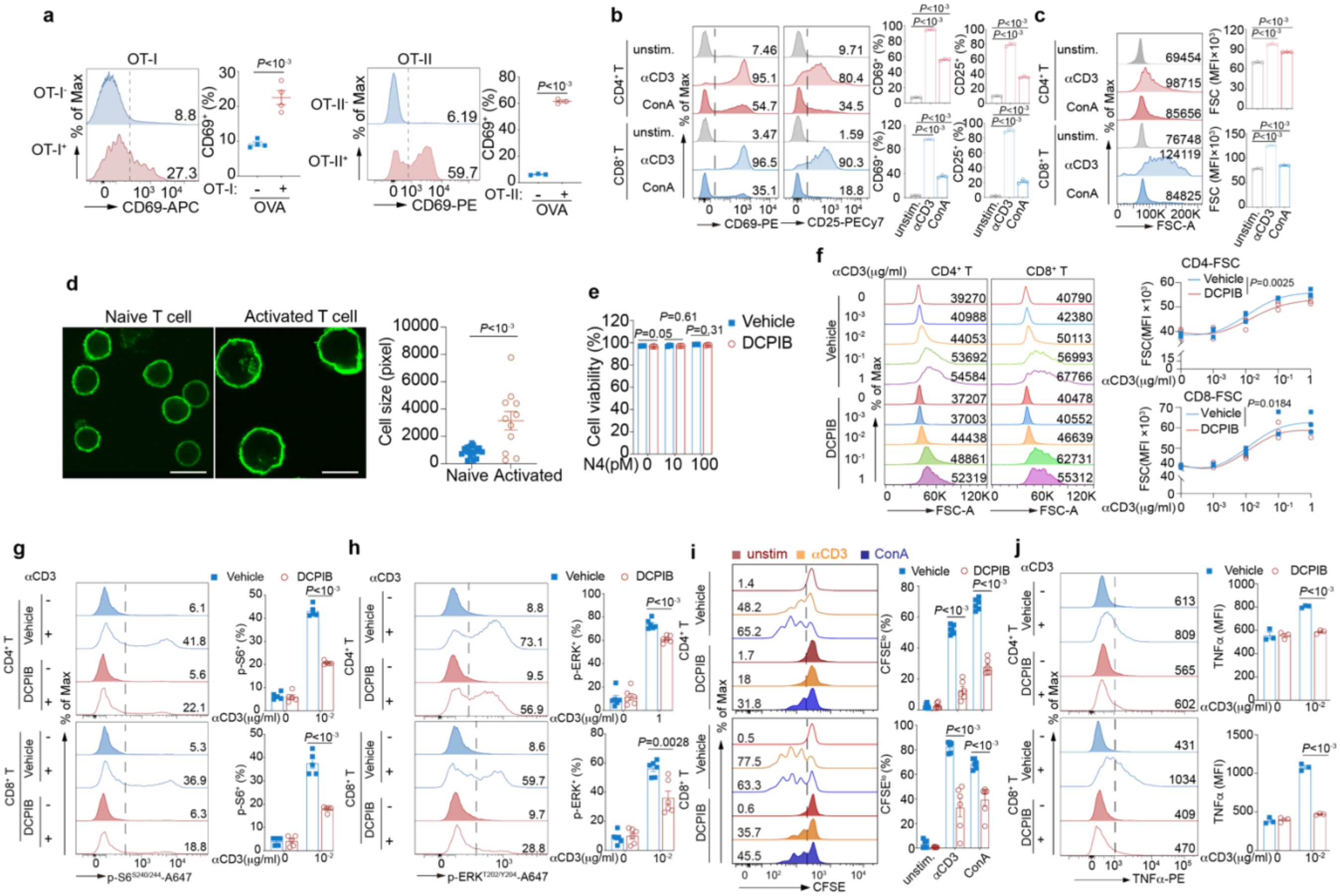

Supplementary Fig. 1. T cell activation and function demand normal VRAC function. a, T cell activation *in vivo*, evaluated by CD69 expression on flow cytometry. OT-I, n=4 mice/group; OT-II, n=3 mice/group. **b**-**c**, T cell activation and cell size measurement *ex vivo*. Splenic CD4^{+} and CD8^{+} T cells obtained from C57 mice spleen were activated by anti-CD3ε (0.1 μg/ml) or ConA (1 μg/ml), and evaluated at 6 hours and 24 hours after stimulation, respectively. n=3 replicates/mouse for **b,c**. **d**, Cell size estimated by confocal microscopy for naïve and activated CD8^{+} T cells (n⩾11). Scale bar: 10 μm. **e**, Effects of DCPIB on cell viability in OT-I CD8^{+} T cells treated with or without DCPIB (25 μM) for 6 hours. DAPI^{+} for dead cells (n=3 replicates/mouse). **f**, Effects of DCPIB on T cell size after activation by anti-CD3ε at indicated concentrations with or without DCPIB (25μM) for 24 hours (n=3 replicates/mouse). **g**-**h**, p-S6$^{S240/244}$ and p-ERK$^{T202/Y204}$ measured by flow cytometry. Splenic CD4^{+} and CD8^{+} T cells with or without DCPIB (25 μM) activated by anti-CD3ε at indicated concentrations for 6 hours (p-S6$^{S240/244}$, n=5 mice/group) and 2 hours (p-ERK$^{T202/Y204}$, n=6 mice/group). **i**, Cell proliferation shown by CFSE dilution. Splenic CD4^{+} and CD8^{+} T cells were labeled with CFSE (1 μM) and activated with anti-CD3ε (0.01 μg/ml) or ConA (0.5 μg/ml) for 48 hours with or without DCPIB (25 μM) (n=6 mice/group). **j**, Production of TNFα in T cells activated with anti-CD3ε (0.1 μg/ml) for 6 hours with or without

DCPIB (25 μM) (n=3 replicates/mouse). Representative flow cytometry histograms (left) and the quantification (right) were shown (**a**-**c** and **f**-**j**). Data are representative of two (**a**,**c**,**d**,**f**), three (**b**,**e**,**j**), or more (**g**-**i**) independent experiments. Unpaired two-sided *t*-test was used in **a**,**d**,**e**,**g**-**j**. One-way ANOVA was in **b**,**c**, and Bonferroni's post-hoc test in **f**. Data are presented as mean ± SEM. Source data are provided as a Source Data file.

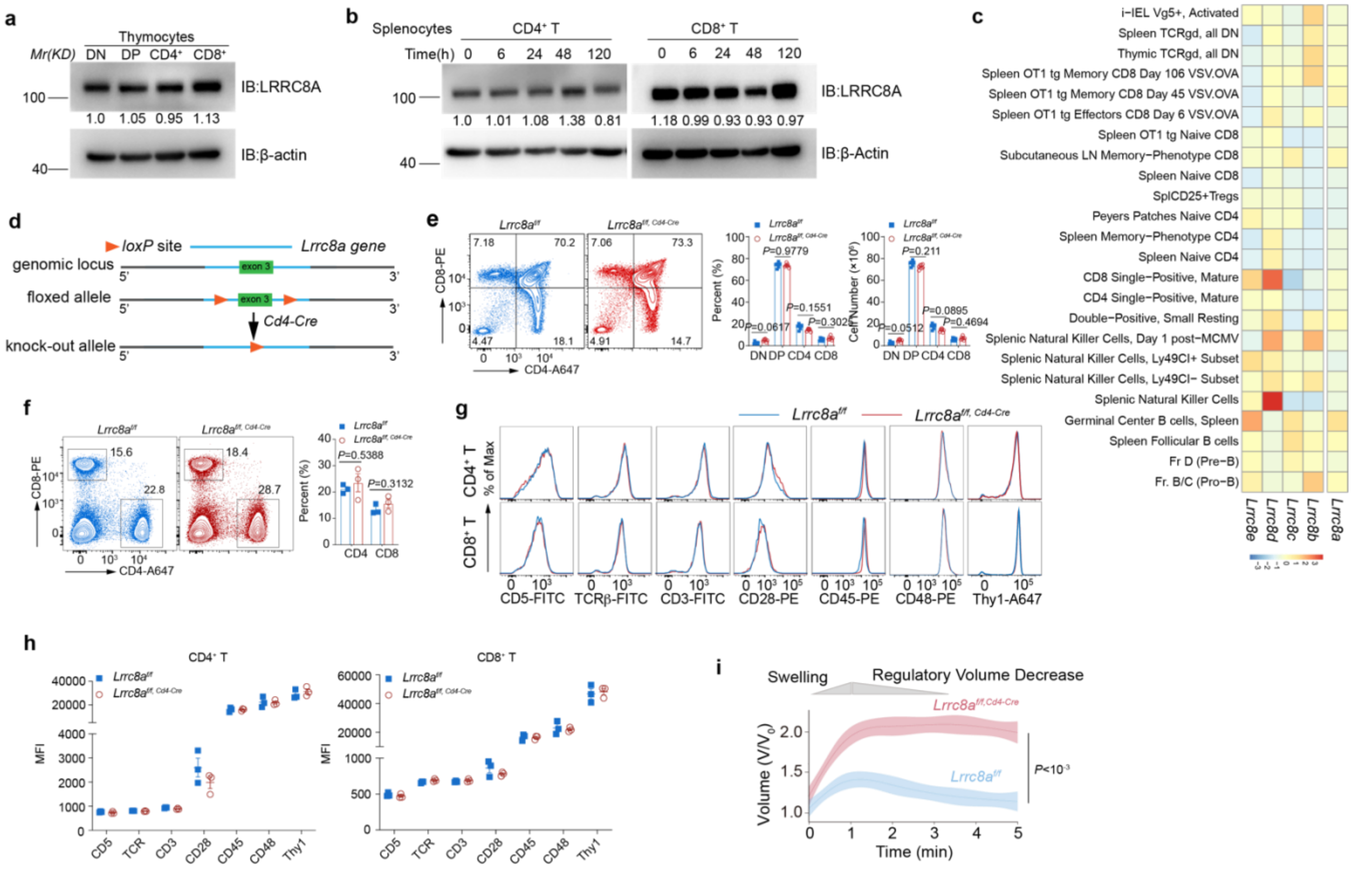

Supplementary Fig. 2. LRRC8A is dispensable for T cell development. a,**b**, Immunoblot of LRRC8A. Thymocyte subsets (double positive DP, double negative DN, $CD4^+$ and $CD8^+$ single positive T cells) were sorted from the thymus (**a**). Purified $CD4^+$ and $CD8^+$ T cells from the spleen of B6 mice activated for indicated time with plate-bound anti-CD3ε (1 μg/ml) and soluble anti-CD28 (1 μg/ml) (**b**). **c**, Expression of *Lrrc8a-e* genes from different lymphocytes obtained from Immunological Genome Project (ImmGen.org). **d**, The strategy for generation of *Lrrc8a* conditional knockout mice. **e**,**f**, T cell subsets in the thymus (**e**) and spleen (**f**) from WT (*Lrrc8a$^{f/f}$*) and cKO (*Lrrc8a$^{f/f,\ Cd4\text{-}Cre}$*) mice. Representative flow cytometry plots (left) and bar graphs (right) showing the percentage and numbers of thymocyte subsets DN, DP, $CD4^+$ and $CD8^+$ single positive T cells (**e**) and splenocyte subsets $CD4^+$ and $CD8^+$ T cells (**f**) (n⩾3 mice/group). **g**,**h**, T cell surface markers expression on $CD4^+$ and $CD8^+$ splenic T cells from WT and cKO mice. Representative histogram of flow cytometry (**g**) and quantification (**h**) were shown (n=3 mice/group). **i**, Relative cell volume (V/V_0) of thymocytes from *Lrrc8a$^{f/f}$* (n≥33 cells) or *Lrrc8a$^{f/f,\ Cd4\text{-}Cre}$* (n≥35 cells) mice in response to acute hypotonic treatment (100 mOsm) measured by microscopy. V_0, volume under isotonic condition. Data are representative of two (**a**,**b**,**g**,**i**), three (**f**) or more (**e**) independent experiments. Two-sided unpaired *t*-test was in **e**,**f**,**h**. Two-way ANOVA with Bonferroni's post-hoc test was in **i**. Data are presented as mean ± SEM. Source data are provided as a Source Data file.

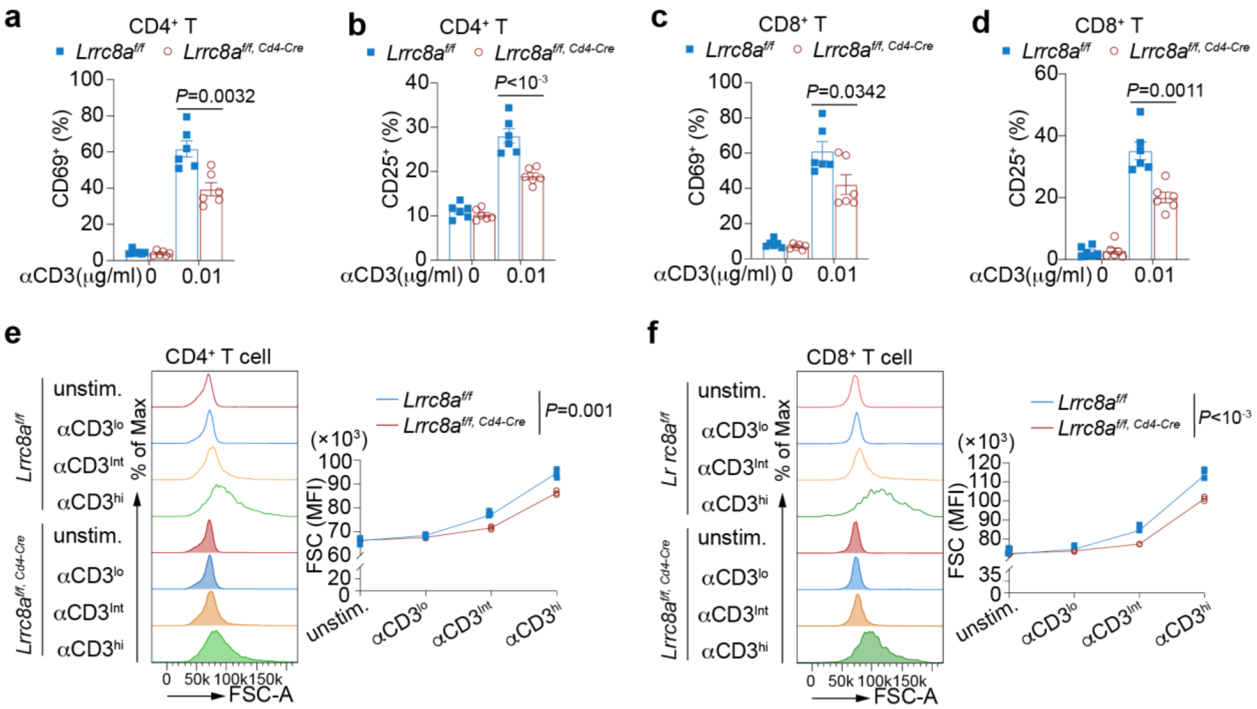

Supplementary Fig. 3. LRRC8A is crucial for T cell blast upon activation. a-d, CD69 and CD25 expression on splenic CD4$^+$ or CD8$^+$ T cells from WT (*Lrrc8a*$^{f/f}$) and conditional KO (cKO, *Lrrc8a*$^{f/f,\ Cd4\text{-}Cre}$) mice, activated by anti-CD3ε at indicated concentration *ex vivo* for 6 hours. Gated on CD4$^+$ or CD8$^+$ T cells (n=6 mice/group). **e**,**f**, T cell blast was monitored by FSC on flow cytometry 24 hours post-stimulation *ex vivo*. Splenic CD4$^+$ (**e**) or CD8$^+$ T cells (**f**) from *Lrrc8a*$^{f/f}$ and *Lrrc8a*$^{f/f,\ Cd4\text{-}Cre}$ mice were activated by anti-CD3ε at indicated concentrations for 24 hours (n=3 replicates/mouse). Antibody concentrations: αCD3^{lo} (0.001 μg/ml), αCD3^{int} (0.01 μg/ml), αCD3^{hi} (0.1 μg/ml). Gated on CD4$^+$ or CD8$^+$ T cells. Data are representative of two (**e**,**f**) or more (**a-d**) independent experiments. Unpaired two-sided *t*-test was used in **a-d**, and two-way ANOVA was in **e**,**f**. Data are presented as mean ± SEM. Source data are provided as a Source Data file.

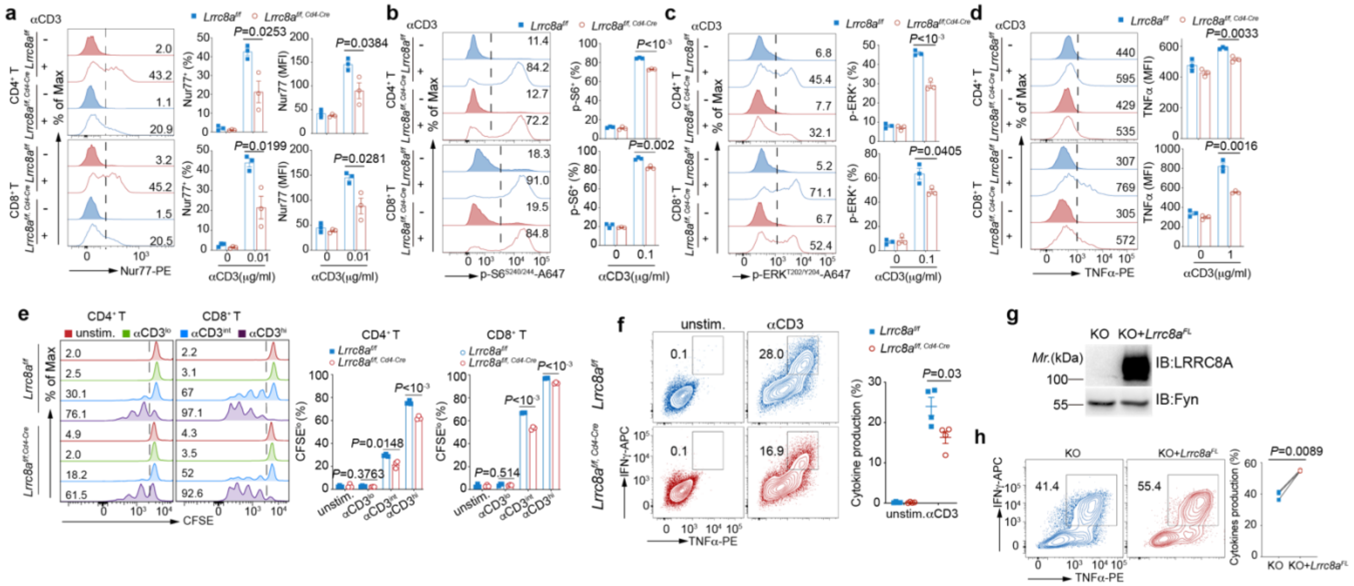

Supplementary Fig. 4. Compromised T cell signaling and functions in loss of LRRC8A. a-d, Nur77 expression (**a**), p-S6$^{S240/244}$ (**b**), p-ERK$^{T202/Y204}$ (**c**), and TNFα production (**d**) were measured by flow cytometry. Splenic CD4^{+} and CD8^{+} T cells from *Lrrc8a*$^{f/f}$ and *Lrrc8a*$^{f/f,\ Cd4\text{-}Cre}$ mice stimulated with anti-CD3ε at indicated concentrations for 6 hours (n=3 replicates/mouse). **e**, *in vitro* T cell proliferation demonstrated by CFSE. CFSE-labeled splenic CD4^{+} and CD8^{+} T cells from *Lrrc8a*$^{f/f}$ and *Lrrc8a*$^{f/f,\ Cd4\text{-}Cre}$ mice were cultured for 2 days in the presence of anti-CD3ε at indicated concentrations (n=3 replicates/mouse). **f**, Cytokines (TNFα and IFNγ) production in previously activated CD8^{+} T cells. CD8^{+} T cells were purified from the spleen of *Lrrc8a*$^{f/f}$ and *Lrrc8a*$^{f/f,\ Cd4\text{-}Cre}$ mice and activated by plate-bound anti-CD3ε (1 μg/ml) and anti-CD28 (1 μg/ml) for 48 hours and expanded in the present with IL-2 (10 U/ml) for another 3 days. Activated T cells were restimulated with plate-bound anti-CD3ε (0.5 μg/ml) for 6 hours, followed by intracellular staining of TNFα and IFNγ (n=4 mice/group). **g**,**h**, LRRC8A protein restoration in LRRC8A KO CD8^{+} T cells (**g**) and TNFα and IFNγ production in KO and KO+*Lrrc8a*^{FL} CD8^{+} T cells (**h**). *Lrrc8a*^{FL}, full-length *Lrrc8a* cDNA. Previously activated CD8^{+} T cells were restimulated with the method in **f** (n=3 replicates/mouse). Representative flow cytometry plot (left) and quantification (right) were shown (**a**-**f**,**h**). Data are representative of two (**a,d**), three (**b**,**c**,**g**,**h**) or four (**e**,**f**) independent experiments. Paired *t*-test was used in (**h**). Two-sided unpaired *t*-test was in **a**-**f**. Data are presented as mean ± SEM. Source data are provided as a Source Data file.

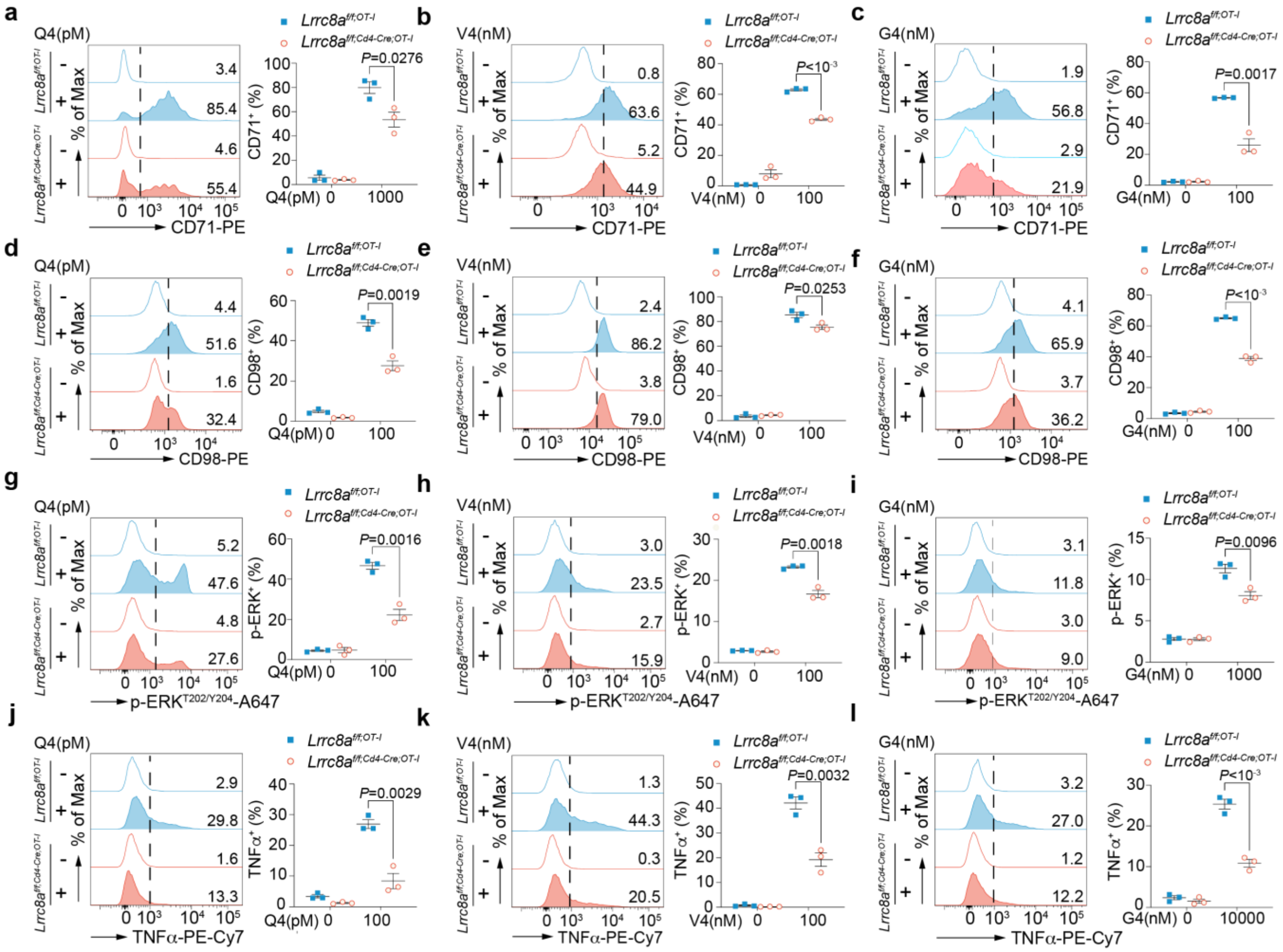

Supplementary Fig. 5. **Defective T cell signaling and function in the absence of LRRC8A.** OT-I $CD8^+$ T cells from WT (*Lrrc8a*$^{f/f;\ OT\text{-}I}$) and LRRC8A KO (*Lrrc8a*$^{f/f,\ Cd4\text{-}Cre;\ OT\text{-}I}$) mice were activated by N4 peptide variants (Q4, V4, G4) at indicated concentrations for 6 hours (CD71/CD98/TNFα) or 2 hours (p-ERK) *ex vivo*. Cells were gated on $TCR\beta^+CD8^+V\alpha2^+$. CD71 and CD98 expression (**a**-**f**), p-ERK1/2 (**g**-**i**), TNFα (**j**-**l**) were examined by flow cytometry. n=3 replicates/mouse in **a**-**l**. Representative flow cytometry plots (left) and the quantification of percentage (right) were shown for all the panels. Data are representative of two (**a**-**l**) independent experiments. Unpaired two-sided *t*-test was used for all the panels. Data are presented as mean ± SEM. Source data are provided as a Source Data file.

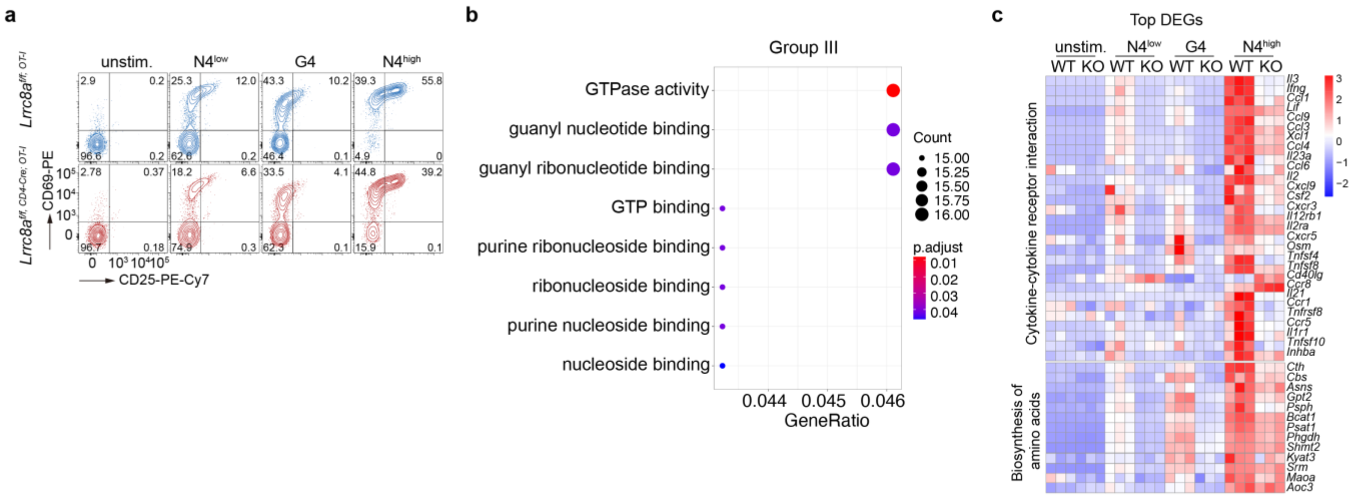

Supplementary Fig. 6. Transcriptome analysis of OT-I CD8$^+$ T cells from WT and LRRC8A KO mice. OT-I CD8$^+$ T cells from WT and LRRC8A KO mice were activated with indicated peptides for 6 hours *ex vivo*, and then sorted for RNA-seq. T cell activation was verified by CD69 and CD25 expression (**a**). N4^{low} (10 pM), G4 (1000 nM), N4^{high} (1000 pM). **b**, GO enrichment analysis of group III genes in Fig. 4**f**. **c**, Heatmap of "Cytokine-cytokine receptor interaction" "Biosynthesis of amino acids" pathway identified by different gene expression (DEGs) analysis.

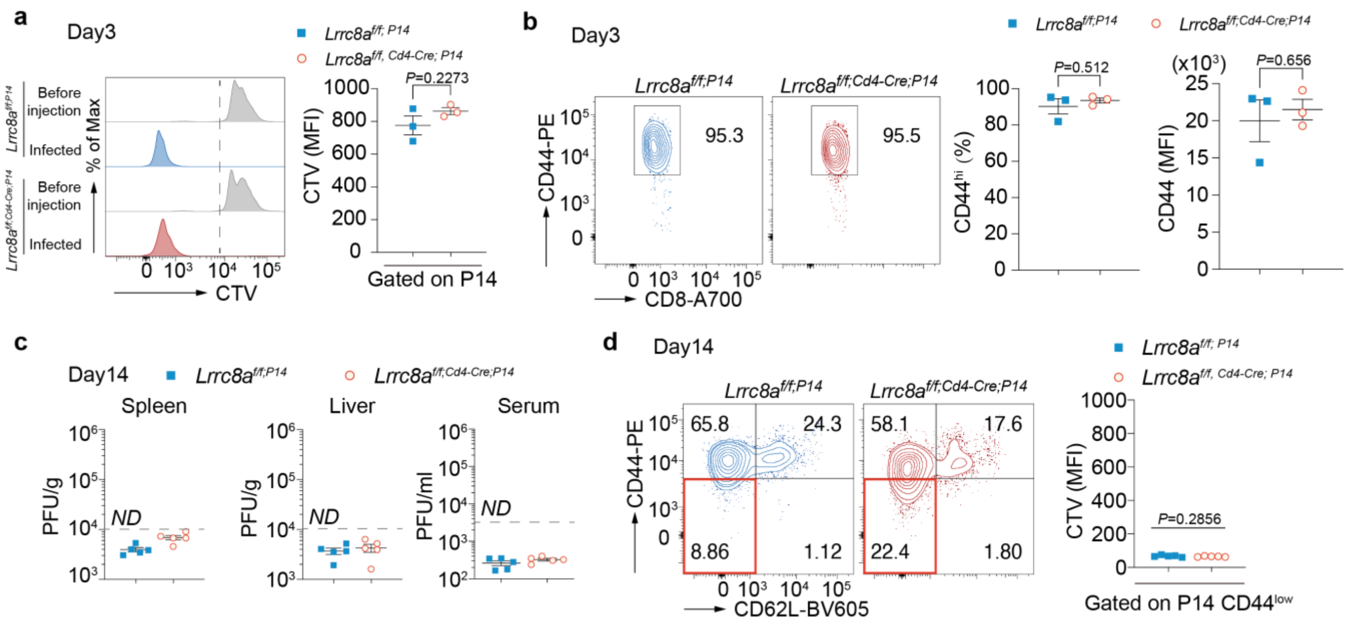

Supplementary Fig. 7. LRRC8A deficiency impairs T cell-mediated antiviral immunity to LCMV Armstrong infection. a,**b**, CellTrace Violet (CTV) (**a**) and CD44 (**b**) expression in P14 T cells were examined after infection for 3 days with LCMV Armstrong as in Fig. 5. n=3 mice/group. **c**, Virus load evaluated in the spleen, liver and serum from mice on day 14 pi (n=5 mice/group). ND, not detected. **d**, CTV in CD44^{low} P14 T cells in the spleen from mice in **c** (n=5 mice/group). Representative flow cytometry plots were shown on the left and their quantification on the right for panels **a,b,d**. Two-sided unpaired *t*-test was used in **a-d**, Data are presented as mean ± SEM. Source data are provided as a Source Data file.

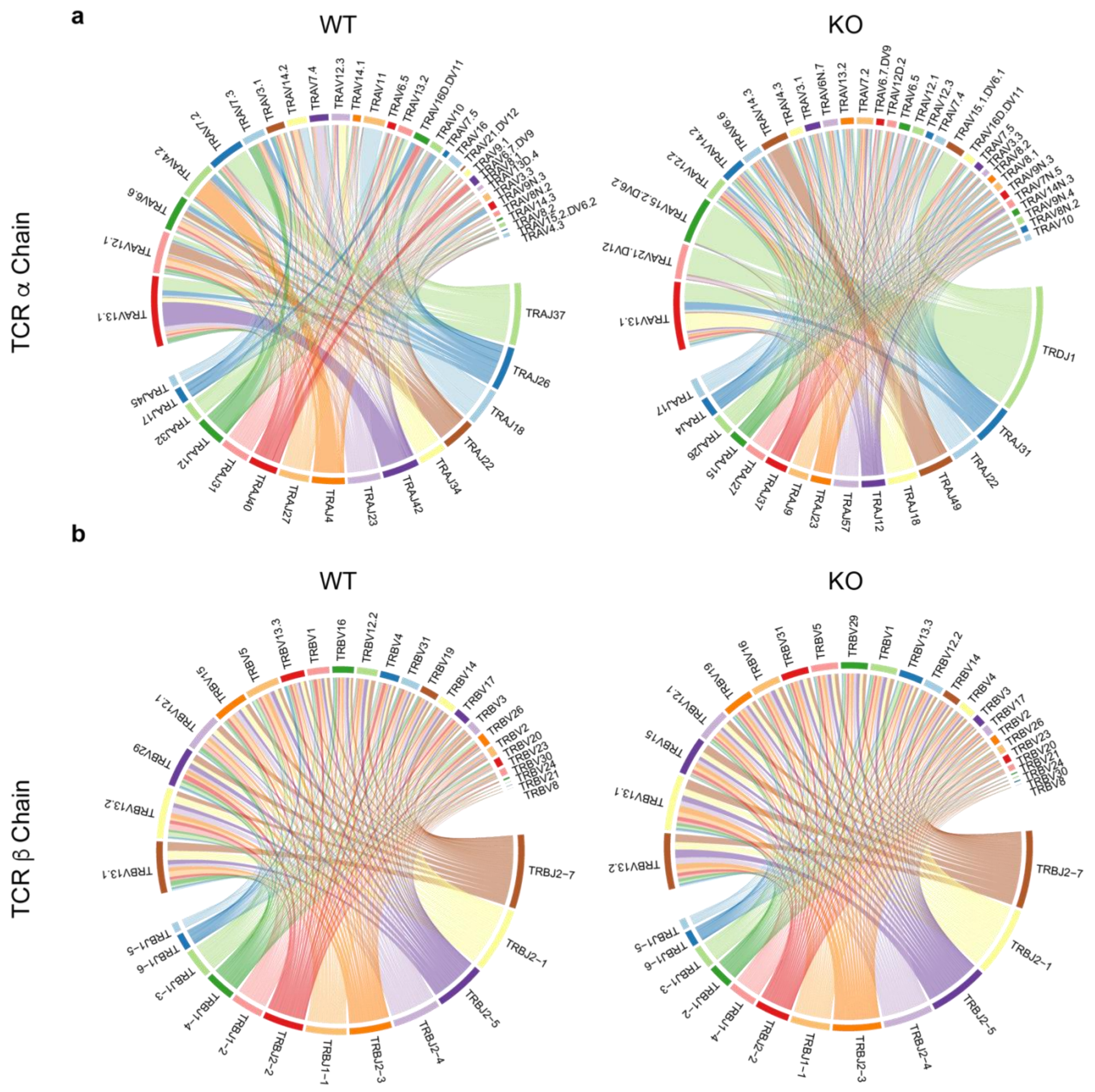

Supplementary Fig. 8. TCR chains rearrangement is unaltered by LRRC8A. a,**b**, Chord diagram showing highly similar V and J rearrangements for both TCR α (**a**) and β (**b**) chains in the thymus from WT (*Lrrc8a*$^{f/f}$) and KO (*Lrrc8a*$^{f/f,\ Cd4\text{-}Cre}$) mice.

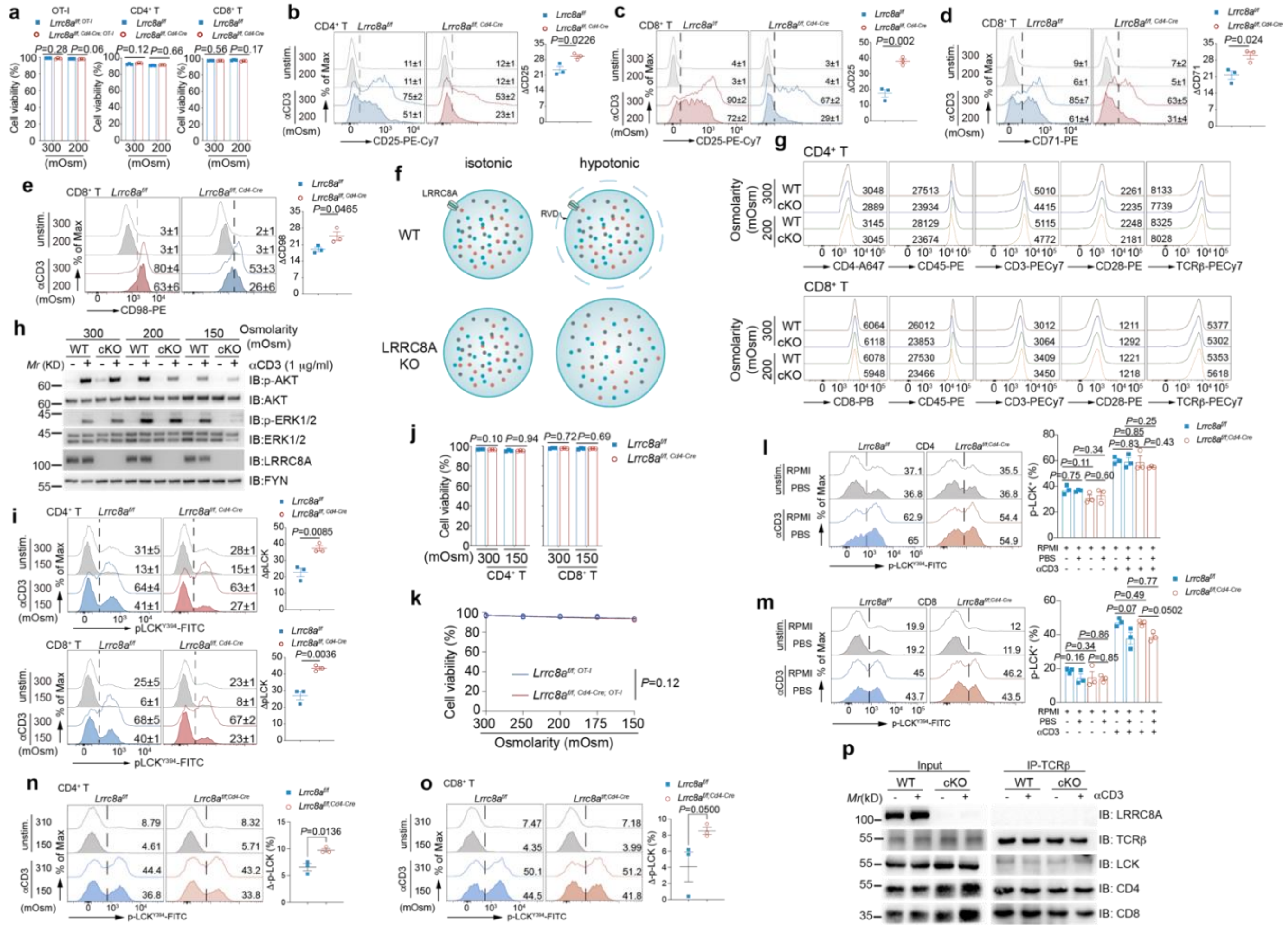

Supplementary Fig. 9. Cell volume regulated by LRRC8A during T cell activation fine-tunes TCR proximal signal. **a**, Cell viability measured by flow cytometry after treatment in isotonic (300 mOsm) or hypotonic (200 mOsm) buffer for 6 hours *ex vivo*. **b**-**e**, Splenic CD4$^+$ and CD8$^+$ T cells from *Lrrc8a*$^{f/f}$ and *Lrrc8a*$^{f/f,\ Cd4\text{-}Cre}$ mice were activated with anti-CD3ε (0.1 μg/ml) under conditions of **a**. The expression of CD25 (**b**,**c**), CD71 (**d**) and CD98 (**e**) were measured by flow cytometry. **f**, Illustration of molecular density changes upon hypotonic cell swelling in WT and KO T cells. **g**, Surface markers expression on splenic CD4$^+$ and CD8$^+$ T cells from WT (*Lrrc8a*$^{f/f}$) and cKO (*Lrrc8a*$^{f/f,\ Cd4\text{-}Cre}$) mice after treated with isotonic (300 mOsm) or hypotonic (150 mOsm) condition. **h**, p-AKT and p-ERK1/2 in previously activated CD8$^+$ T cells stimulated with anti-CD3ε (1 μg/ml) for 2 minutes. **i**, p-LCK^{Y394} of splenic CD4$^+$ and CD8$^+$ T cells from *Lrrc8a*$^{f/f}$ and *Lrrc8a*$^{f/f,\ Cd4\text{-}Cre}$ mice measured by flow cytometry. Splenic T cells were activated by anti-CD3ε (1 μg/ml) *ex vivo* for 30 minutes under isotonic (300 mOsm) or hypotonic (150 mOsm) conditions. **j**,**k**, Effects of hypotonicity on cell viability measured by flow cytometry in CD4$^+$ and CD8$^+$ T, and OT-I CD8$^+$ T cells at indicated osmolarity. **l**-**o**, p-LCK^{Y394} of splenic CD4$^+$ and CD8$^+$ T cells from *Lrrc8a*$^{f/f}$ and *Lrrc8a*$^{f/f,\ Cd4\text{-}Cre}$ mice measured by flow cytometry. Splenic T cells were activated by anti-CD3ε (1 μg/ml) *ex vivo* for 30 minutes in the indicated solutions. RPMI was mixed with or without PBS at the ratio of 1:1 in **l**,**m**, and the buffer osmolarity adjusted using mannitol in

n,**o** (n=3 replicates/mouse for **a**-**e**, **i**-**o**). **p**, Immunoprecipitation of TCRβ and followed by immunoblot of LRRC8A and other key molecules in TCR complex in the thymocytes. Representative histogram (left) and the quantifications (*eg*. $\Delta CD25 = CD25_{300\ mOsm} - CD25_{200\ mOsm}$) were shown for **b**-**e**, and **i**. Data are representative of two (**g**-**k,p**) or three (**a**-**e,l**-**o**) independent experiments. Unpaired two-sided *t*-test was used in **a**-**e**, **i**-**j**, **l**-**o**. Two-way ANOVA was in **k**. Data are presented as mean ± SEM. Source data are provided as a Source Data file.

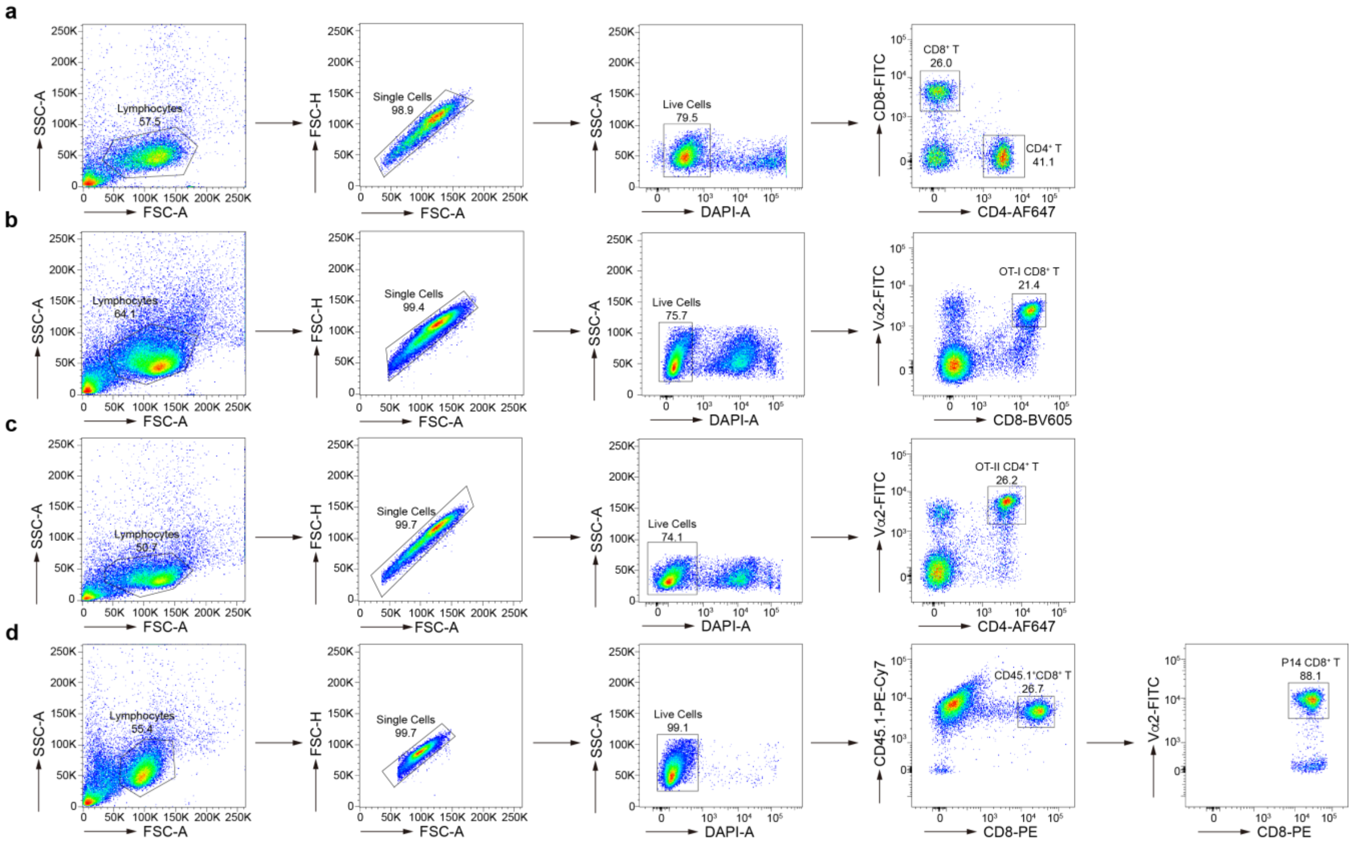

Supplementary Fig. 10. Gating strategies of flow cytometry. (**a**-**d**) Gating strategies of $CD4^+$ and $CD8^+$ T cells (**a**), OT-I $CD8^+$ T cells (**b**), OT-II $CD4^+$ T cells (**c**) and P14 $CD8^+$ T cells (**d**).
