# Supplementary material for: Cell volume controlled by LRRC8A-formed volume-regulated anion channels fine-tunes T cell activation and function
Source: Nat Commun. 2023 Nov 4;14:7075. doi: 10.1038/s41467-023-42817-y (PMC10625614; doi:10.1038/s41467-023-42817-y)
Supplement: Supplementary file 3 — Reporting Summary [file 41467_2023_42817_MOESM3_ESM.pdf]

nature portfolio

Corresponding author(s): Ning Wu, Zheng Liu and Lei Yin

Last updated by author(s): Sep 27,2023

Reporting Summary

Nature Portfolio wishes to improve the reproducibility of the work that we publish. This form provides structure for consistency and transparency in reporting. For further information on Nature Portfolio policies, see our Editorial Policies and the Editorial Policy Checklist.

Statistics

For all statistical analyses, confirm that the following items are present in the figure legend, table legend, main text, or Methods section.

n/a	Confirmed	
☐	☒	The exact sample size (*n*) for each experimental group/condition, given as a discrete number and unit of measurement
☐	☒	A statement on whether measurements were taken from distinct samples or whether the same sample was measured repeatedly
☐	☒	The statistical test(s) used AND whether they are one- or two-sided<br>*Only common tests should be described solely by name; describe more complex techniques in the Methods section.*
☒	☐	A description of all covariates tested
☒	☐	A description of any assumptions or corrections, such as tests of normality and adjustment for multiple comparisons
☐	☒	A full description of the statistical parameters including central tendency (e.g. means) or other basic estimates (e.g. regression coefficient) AND variation (e.g. standard deviation) or associated estimates of uncertainty (e.g. confidence intervals)
☐	☒	For null hypothesis testing, the test statistic (e.g. *F*, *t*, *r*) with confidence intervals, effect sizes, degrees of freedom and *P* value noted<br>*Give P values as exact values whenever suitable.*
☒	☐	For Bayesian analysis, information on the choice of priors and Markov chain Monte Carlo settings
☒	☐	For hierarchical and complex designs, identification of the appropriate level for tests and full reporting of outcomes
☒	☐	Estimates of effect sizes (e.g. Cohen's *d*, Pearson's *r*), indicating how they were calculated

Our web collection on statistics for biologists contains articles on many of the points above.

Software and code

Policy information about availability of computer code

Data collection	Western blots:Bio-Rad ChemiDocTM Touch Imaging System<br>Confocal microscopy: Olympus FV3000<br>Flow-Cytometry:BD Fortessa cell analyzer<br>TCR sequencing: Illumina NovaSeq 6000 platform<br>DNA sequencing: DNBSEQ-T7
Data analysis	Statistics and graph production:Prism, Graphpad (v9)<br>Flow cytometry:FlowJo (v10.5)<br>RNA seq data analyze: R (4.1.3)<br>Protein quantification:ImageJ (1.53)

For manuscripts utilizing custom algorithms or software that are central to the research but not yet described in published literature, software must be made available to editors and reviewers. We strongly encourage code deposition in a community repository (e.g. GitHub). See the Nature Portfolio guidelines for submitting code & software for further information.

Data

Policy information about availability of data

All manuscripts must include a data availability statement. This statement should provide the following information, where applicable:

- Accession codes, unique identifiers, or web links for publicly available datasets
- A description of any restrictions on data availability
- For clinical datasets or third party data, please ensure that the statement adheres to our policy

Datasets generated by RNA and TCR sequencing in this study have been deposited in the NCBI Gene Expression Omnibus (GEO) database under the accession number GSE243985 and GSE243574. Source data are provided with this paper.

Human research participants

Policy information about studies involving human research participants and Sex and Gender in Research.

Reporting on sex and gender	N/A
Population characteristics	N/A
Recruitment	N/A
Ethics oversight	N/A

Note that full information on the approval of the study protocol must also be provided in the manuscript.

Field-specific reporting

Please select the one below that is the best fit for your research. If you are not sure, read the appropriate sections before making your selection.

☒ Life sciences ☐ Behavioural & social sciences ☐ Ecological, evolutionary & environmental sciences

For a reference copy of the document with all sections, see nature.com/documents/nr-reporting-summary-flat.pdf

Life sciences study design

All studies must disclose on these points even when the disclosure is negative.

Sample size	The sample size and statistical analyses are described in the relevant Figure legends. No statistical methods were used to predetermine sample size. Sample sizes were chosen to reliably determine the differences between groups. Independent experiments were performed with sufficient biological replicates according to or exceeding standards in the field.
Data exclusions	No data were excluded from analyses.
Replication	Each experiment was repeated at least two independent times. All experimental repeats were successful.
Randomization	Allocation of test animals used in our study was random. There was no targeted selection of individual animals for specific treatments.
Blinding	Blinding was not used in our study. WT and KO mice were chosen for experiments based on their genotypes after weaning. All experimental manipulations were performed in parallel by following the same protocol to avoid bias.

Reporting for specific materials, systems and methods

We require information from authors about some types of materials, experimental systems and methods used in many studies. Here, indicate whether each material, system or method listed is relevant to your study. If you are not sure if a list item applies to your research, read the appropriate section before selecting a response.

Materials & experimental systems

n/a	Involved in the study
	☒ Antibodies
	☒ Eukaryotic cell lines
☒	☐ Palaeontology and archaeology
	☒ Animals and other organisms
☒	☐ Clinical data
☒	☐ Dual use research of concern

Methods

n/a	Involved in the study
☒	☐ ChIP-seq
	☒ Flow cytometry
☒	☐ MRI-based neuroimaging

Antibodies

Antibodies used

For flow sytometry:
Alexa Fluor® 647 anti-mouse CD90.2 (Thy1.2) Antibody(30-H12,105318,Biolegend,1:500)
Alexa Fluor® 700 anti-mouse CD8a Antibody (53-6.7,100730,Biolegend,1:1000)
APC anti-mouse CD127 (IL-7Rα) Antibody (SB/199,121122,Biolegend,1:500)
APC anti-mouse IFN-γ Antibody (XMG1.2,505810,Biolegend,1:500)
APC/Cyanine7 anti-mouse TNF-α Antibody (MP6-XT22,506344,Biolegend,1:500)
BD Pharmingen™ FITC Rat Anti-Mouse CD5 (53-7.3,553020,BD,1:500)
Brilliant Violet 605™ anti-mouse CD62L Antibody (MEL-14,104438,Biolegend,1:500)
CD25-PECy7(PC61,102016,Biolegend,1:500)
CD28 Monoclonal Antibody (37.51), Biotin, eBioscience™ (37.51,13-0281-85,Invitrogen,1:500)
CD3-FITC(17A2， 100204， Biolegend,1:500)
CD4-AF647 (GK1.5， sc-13573， Santa Cruz,1:1000)
CD98 Monoclonal Antibody (RL388), PE, eBioscience™(RL3188， 12-0981-81， Invitrogen,1:500)
Cell trace violite (C34557， invitrogen,1:1000)
CFSE (C34554， invitrogen,1:1000)
DAPI(D9542， Sigma,1:100)
F(ab')2-Goat anti-Mouse IgG (H+L) Secondary Antibody, FITC, eBioscience™ (11-4010-82， invitrogen,1:500)
FITC anti-mouse TCR Vα2 Antibody(B20.1,127806,Biolegend,1:500)
FITC anti-mouse TCR β chain Antibody (H57-597,109206, Biolegend,1:500)
Goat anti-Rabbit IgG (H+L) Cross-Adsorbed Secondary Antibody, Alexa Fluor™ 647 (A21244, Invitrogen,1:500)
Nur77 Monoclonal Antibody (12.14), PE, eBioscience™ (12.14,12-5965-82,Invitrogen,1:500)
Pacific Blue™ anti-mouse CD8a Antibody (53-6.7,100725,Biolegend,1:1000)
PE anti-mouse CD45 Antibody (30-F11,103106,Biolegend,1:500)
PE anti-mouse CD48 Antibody (HM48-1,103406,Biolegend,1:500)
PE anti-mouse CD69 Antibody (H1.2F3,104508,Biolegend,1:500)
PE anti-mouse CD71 Antibody (R17217,113807,Biolegend,1:500)
PE anti-mouse CD8a Antibody (53-6.7,100708,Biolegend,1:1000)
PE anti-mouse Ki-67 Antibody (16A8,652404,Biolegend,1:500)
PE anti-mouse/human CD44 Antibody (IM7,103008,Biolegend,1:500)
PE anti-mouse/human KLRG1 (MAFA) Antibody (2F1/KLRG1,138408, Biolegend,1:500)
PE Streptavidin (405204, Biolegend,1:500)
PE/Cyanine7 anti-mouse CD3 Antibody(17A2,100220,Biolegend,1:500)
PE/Cyanine7 anti-mouse CD45.1 Antibody (A20,110730,Biolegend,1:500)
PE/Cyanine7 anti-mouse TCR β chain Antibody(H57-597,109222,Biolegend,1:500)
Phospho-p44/42 MAPK (Erk1/2) (E10,9106,CST,1:100)
Phospho-S6 Ribosomal Protein (Ser240/244) (D68F8,5364,CST,1:100)
Phospho-ZAP70/Syk (Tyr319, Tyr352) Recombinant Rabbit Monoclonal Antibody(Zap70Y319-A3 MA5-28069 Invitrogen,1:100)
Purified anti-LAT Phospho (Tyr171) Antibody (A20005D 946602 Biolegend,1:100)
Purified anti-Lck Phospho (Tyr394) Antibody (A8002D 933102 Biolegend,1:100)
For western blot:
Recombinant Anti-LRRC8A antibody(ab254389, Abcam,1:100)
beta Actin Antibody (C4) (sc-47778,Santa Cruz,1:100)
Lck Antibody(2752,CST,1:100)
FYN(4023,CST,1:100)
GAPDH Polyclonal antibody(10494-1-AP,Proteintech,1:100)
pTyr(4G10,05-1050,Merk Millipore,1:1000)
ZAP70 Monoclonal antibody(1A11G9,60200-1,Peoteintech,1:100)
LAT Polyclonal antibody (11326-1-AP,Peoteintech,1:100)
Purified anti-mouse TCR β chain Recombinant Antibody(159702,Biolegend,1:100)
CD3z(12837-2-AP,Proteintech,1:100)
Purified anti-mouse CD4 Antibody(100402,Biolegend,1:100)
Purified anti-mouse CD8a Antibody(100702,Biolegend,1:100)
Phalloidin-iFluor 488 Reagent (ab176753,Abcam,1:100)

Phospho-Akt (Ser473) (D9E) XP® Rabbit mAb D9E(4060,CST,1:100)
Akt (pan) (C67E7) Rabbit mAb(C67E7,4691S,CST,1:100)
ERK(ABS44,Merk Millipore,1:100)

Validation

Antibodies used have been validated by the manufacturers according to their websites. Detailed info 「mation are available on their vendor websites.
Alexa Fluor® 647 anti-mouse CD90.2 (Thy1.2) Antibody (https://www.biolegend.com/en-us/products/alexa-fluor-647-anti-mouse-cd90-2-thy1-2-antibody-3125)
Alexa Fluor® 700 anti-mouse CD8a Antibody(https://www.biolegend.com/en-us/products/alexa-fluor-700-anti-mouse-cd8a-antibody-3387)
APC anti-mouse CD127 (IL-7Rα) Antibody(https://www.biolegend.com/en-us/products/apc-anti-mouse-cd127-il-7ralpha-antibody-5228)
APC anti-mouse IFN-γ Antibody(https://www.biolegend.com/en-us/products/apc-anti-mouse-ifn-gamma-antibody-993)
APC/Cyanine7 anti-mouse TNF-α Antibody(https://www.biolegend.com/en-us/products/apc-cyanine7-anti-mouse-tnf-alpha-antibody-12117)
BD Pharmingen™ FITC Rat Anti-Mouse CD5 (https://www.bdbiosciences.com/en-us/products/reagents/flow-cytometry-reagents/research-reagents/single-color-antibodies-ruo/fitc-rat-anti-mouse-cd5.553020)
Brilliant Violet 605™ anti-mouse CD62L Antibody (https://www.biolegend.com/en-us/products/brilliant-violet-605-anti-mouse-cd62l-antibody-7687)
CD25-PECy7 (https://www.biolegend.com/en-us/products/pe-cyanine7-anti-mouse-cd25-antibody-1929)
CD28 Monoclonal Antibody (37.51), Biotin, eBioscience™ (https://www.thermofisher.com/antibody/product/CD28-Antibody-clone-37-51-Monoclonal/13-0281-85)
CD3-FITC (https://www.biolegend.com/en-us/products/fitc-anti-mouse-cd3-antibody-45)
CD4-AF647 (https://www.scbt.com/p/cd4-antibody-gk1-5?requestFrom=search)
CD98 Monoclonal Antibody (RL388), PE, eBioscience™ (https://www.thermofisher.com/antibody/product/CD98-Antibody-clone-RL388-Monoclonal/12-0981-81)
Cell trace violite (https://www.thermofisher.com/order/catalog/product/C34557)
CFSE (https://www.thermofisher.com/order/catalog/product/C34570?SID=srch-srp-C34570)
DAPI (https://www.sigmaaldrich.com/HK/zh/product/sigma/d9542)
F(ab')2-Goat anti-Mouse IgG (H+L) Secondary Antibody, FITC, eBioscience™ (https://www.thermofisher.com/antibody/product/Goat-anti-Mouse-IgG-H-L-Secondary-Antibody-Polyclonal/11-4010-82)
FITC anti-mouse TCR Vα2 Antibody(https://www.biolegend.com/en-us/products/fitc-anti-mouse-tcr-valpha2-antibody-4804)
FITC anti-mouse TCR β chain Antibody(https://www.biolegend.com/en-us/products/fitc-anti-mouse-tcr-beta-chain-antibody-270)
Goat anti-Rabbit IgG (H+L) Cross-Adsorbed Secondary Antibody, Alexa Fluor™ 647 (https://www.thermofisher.com/antibody/product/Goat-anti-Rabbit-IgG-H-L-Cross-Adsorbed-Secondary-Antibody-Polyclonal/A-21244)
Nur77 Monoclonal Antibody (12.14), PE, eBioscience™ (https://www.thermofisher.com/antibody/product/Nur77-Antibody-clone-12-14-Monoclonal/12-5965-82)
Pacific Blue™ anti-mouse CD8a Antibody (https://www.biolegend.com/en-us/products/pacific-blue-anti-mouse-cd8a-antibody-2856)
PE anti-mouse CD45 Antibody (https://www.biolegend.com/en-us/products/pe-anti-mouse-cd45-antibody-100)
PE anti-mouse CD48 Antibody (https://www.biolegend.com/en-us/products/pe-anti-mouse-cd48-antibody-293)
PE anti-mouse CD69 Antibody (https://www.biolegend.com/en-us/products/pe-anti-mouse-cd69-antibody-265)
PE anti-mouse CD71 Antibody (https://www.biolegend.com/en-us/products/pe-anti-mouse-cd71-antibody-1631)
PE anti-mouse CD8a Antibody (https://www.biolegend.com/en-us/products/pe-anti-mouse-cd8a-antibody-155)
PE anti-mouse Ki-67 Antibody (https://www.biolegend.com/en-us/products/pe-anti-mouse-ki-67-antibody-8134)
PE anti-mouse/human CD44 Antibody (https://www.biolegend.com/en-us/products/pe-anti-mouse-human-cd44-antibody-2206)
PE anti-mouse/human KLRG1 (MAFA) Antibody (https://www.biolegend.com/en-us/products/pe-anti-mouse-human-klrg1-mafa-antibody-6593)
PE Streptavidin (https://www.biolegend.com/en-us/products/pe-streptavidin-1475)
PE/Cyanine7 anti-mouse CD3 Antibody (https://www.biolegend.com/en-us/products/pe-cyanine7-anti-mouse-cd3-antibody-6060)
PE/Cyanine7 anti-mouse CD45.1 Antibody (https://www.biolegend.com/en-us/products/pe-cyanine7-anti-mouse-cd45-1-antibody-4917)
PE/Cyanine7 anti-mouse TCR β chain Antibody (https://www.biolegend.com/en-us/products/pe-cyanine7-anti-mouse-tcr-beta-chain-antibody-4144)
Phospho-p44/42 MAPK (Erk1/2) (https://www.cellsignal.cn/products/primary-antibodies/phospho-p44-42-mapk-erk1-2-thr202-tyr204-e10-mouse-mab/9106?site-search-type=Products&N=4294956287&Ntt=e10&fromPage=plp)
Phospho-S6 Ribosomal Protein (Ser240/244) (https://www.cellsignal.cn/products/primary-antibodies/phospho-s6-ribosomal-protein-ser240-244-d68f8-xp-rabbit-mab/5364?site-search-type=Products&N=4294956287&Ntt=5364&fromPage=plp&_requestid=3728569)
Phospho-ZAP70/Syk (Tyr319, Tyr352) Recombinant Rabbit Monoclonal Antibody (https://www.thermofisher.com/antibody/product/Phospho-ZAP70-Syk-Tyr319-Tyr352-Antibody-clone-Zap70Y319-A3-Recombinant-Monoclonal/MA5-28069)
Purified anti-LAT Phospho (Tyr171) Antibody (https://www.biolegend.com/en-us/products/purified-anti-lat-phospho-tyr171-antibody-21505)
Purified anti-Lck Phospho (Tyr394) Antibody (https://www.biolegend.com/en-us/products/purified-anti-lck-phospho-tyr394-antibody-18469)
Recombinant Anti-LRRC8A antibody(https://www.abcam.com/products/primary-antibodies/lrrc8a-antibody-epr23402-29-ab254389.html)
beta Actin Antibody (C4) (https://www.scbt.com/p/beta-actin-antibody-c4?requestFrom=search)
Lck Antibody(https://www.cellsignal.cn/products/primary-antibodies/lck-antibody/2752?site-search type=Products&N=4294956287&Ntt=2752&fromPage=plp&_requestid=3729151)

FYN(https://www.cellsignal.cn/products/primary-antibodies/fyn-antibody/4023?site-searchtype=Products&N=4294956287&Ntt=4023&fromPage=plp&_requestid=3729300)
GAPDH Polyclonal antibody (https://www.ptglab.com/products/GAPDH-Antibody-10494-1-AP.htm)
pTyr(https://www.emdmillipore.com/US/en/product/Anti-Phosphotyrosine-Antibody-4G10-Platinum,MM_NF-05-1050X)
ZAP70 Monoclonal antibody(https://www.ptglab.com/products/ZAP70-Antibody-60200-1-Ig.htm)
LAT Polyclonal antibody (https://www.ptglab.com/products/LAT-Antibody-11326-1-AP.htm)
Purified anti-mouse TCR β chain Recombinant Antibody(https://www.biolegend.com/en-us/products/purified-anti-mouse-tcr-beta-chain-recombinant-antibody-20016)
CD3z(https://www.ptglab.com/products/CD247-Antibody-12837-2-AP.htm)
Purified anti-mouse CD4 Antibody(https://www.biolegend.com/en-us/products/purified-anti-mouse-cd4-antibody-252)
Purified anti-mouse CD8a Antibody(https://www.biolegend.com/en-us/products/purified-anti-mouse-cd8a-antibody-157)
Phalloidin-iFluor 488 Reagent (https://www.abcam.com/products/chip-kits/phalloidin-ifluor-488-reagent-ab176753.html)
Phospho-Akt (Ser473) (D9E) XP® Rabbit mAb (https://www.cellsignal.cn/products/primary-antibodies/phospho-akt-ser473-d9e-xp-rabbit-mab/4060?site-search-type=Products&N=4294956287&Ntt=%234060&fromPage=plp&_requestid=3739214)
Akt (pan) (C67E7) Rabbit mAb (https://www.cellsignal.cn/products/primary-antibodies/akt-pan-c67e7-rabbit-mab/4691?site-search-type=Products&N=4294956287&Ntt=4691&fromPage=plp&_requestid=3739097)
ERK (https://www.emdmillipore.com/US/en/product/Anti-MAPK-1-2-Antibody,MM_NF-ABS44)

Eukaryotic cell lines

Policy information about cell lines and Sex and Gender in Research

Cell line source(s)	HEK293T (CRL-11268) cell line is gift from the Dr. Weimin Wang (Huazhong University of Science and Technology) who obtained from ATCC.
Authentication	Cell lines were not authenticated.
Mycoplasma contamination	The cell line is tested for mycoplasma contamination using a PCR-based method. And the cells used in this study are negative for mycoplasma.
Commonly misidentified lines (See ICLAC register)	NA

Animals and other research organisms

Policy information about studies involving animals; ARRIVE guidelines recommended for reporting animal research, and Sex and Gender in Research

Laboratory animals	B6.SJL-Ptprca Pepcb/BoyJ(CD45.l), B6;D2-Tg(TcrLCMV)327Sdz/JDvsJ(P14), C57BL/6-Tg (TcraTcrb) 1100Mjb/J (OT-1), B6.Cg-Tg (TcraTcrb)425Cbn/J(OT-II) mice were obtained from the Jackson Laboratory. Lrrc8a floxed mice with C57BL/6 background we generated in this study.<br>Male and female mice were maintained in specific pathogen free (SPF) animal facility and sacrificed for experiments at the age of 6-12 weeks. All animal experimentation was approved by the Animal Care Committee of Huazhong University of Science and Technology.<br>All mice were housed in individually ventilated cages (IVC) with a maximum of 5 mice/cage. The relative humidity ranged between 40-60% and temperature at 18-23°C. Mice were exposed to a 12-hour light-dark cycle. This has been described in in the 'Methods' section of the manuscript under 'mice'.
Wild animals	The study did not involve the use of wild animals.
Reporting on sex	Both male and female mice were used in this study with same results. Age- and sex-matched mice were used for experimentation
Field-collected samples	Not applicable
Ethics oversight	All animal experiments were performed under protocols approved by the Animal Care and Use Committee of School of Basic Medicine, Tongji Medical College, Huazhong University of Science and Technology.

Note that full information on the approval of the study protocol must also be provided in the manuscript.

Flow Cytometry

Plots

Confirm that:

- [x] The axis labels state the marker and fluorochrome used (e.g. CD4-FITC).
- [x] The axis scales are clearly visible. Include numbers along axes only for bottom left plot of group (a 'group' is an analysis of identical markers).
- [x] All plots are contour plots with outliers or pseudocolor plots.
- [x] A numerical value for number of cells or percentage (with statistics) is provided.

Methodology

Sample preparation	Single cell suspension from mouse organs were freshly isolated using lympholyte (Tebu-Bio)
Instrument	Data were acquired on a Fortessa (BD Biosciences) or cells were sorted on Fortessa (BD Biosciences)
Software	FlowJo (V10.5)
Cell population abundance	Cells were isolated at >=95% purity as assess on FACS.
Gating strategy	For all samples, cells were first gated on singlets and then by the viability marker to gate live cells. T cells were marked as CD4+ or CD8+. For OT-I , OT-II mice, T cells marked as CD8+Vα2+ or CD4+ Vα2+. For P14 mice, T cells marked as CD45.1+CD8+Vα2+.

- [x] Tick this box to confirm that a figure exemplifying the gating strategy is provided in the Supplementary Information.
